# A Rare Case of Bladder Perineurioma

**DOI:** 10.7759/cureus.25938

**Published:** 2022-06-14

**Authors:** Germano José Ferraz de Arruda, Diego Rodrigo Dametto, Thiago da Silveira Antoniassi, José Germano Ferraz de Arruda, Fernando Nestor Facio

**Affiliations:** 1 Urology, Faculdade de Medicina de São José do Rio Preto, Sao Jose do Rio Preto, BRA; 2 Urology, Faculdade de Medicina de São José do Rio Preto, São José do Rio Preto, BRA

**Keywords:** case report, nerve sheath neoplasms, bladder neoplasms, bladder tumors, perineurioma

## Abstract

Perineuriomas are benign, rare neoplasms of the nerve sheath, basically in two forms: intraneural and extraneural. Extraneural forms are mainly found in the trunk and limbs, while visceral organs are rarely affected. To date, there have been no previous reports in the literature of bladder perineurioma. In this case, we report a young adult with hematuria and bladder tumor which after surgical resection and immunohistochemical study was confirmed to be a perineurioma. Therefore, this should be included in the differential diagnosis during the analysis of resections of bladder tumors.

## Introduction

Perineurioma was first described by Lazarus and Trombetta in 1978, as a rare group of benign neoplasms of the nerve sheath, being composed exclusively of cells with perineural differentiation. They represent approximately 1% of all soft tissue neoplasms [1,2]. There are about 200 cases described in the literature, which are slightly more common in adult women [3].

Perineuriomas basically appear in two main forms: intraneural, restricted to the limits of peripheral nerves, and extraneural, mainly found in the skin and soft tissues of the trunk and limbs [4]. Head, neck, and visceral organs are rarely affected [3], and there are no reports in the literature that describe bladder perineurioma.

## Case presentation

This is the case of a 29-year-old patient, without previous comorbidities and no use of medications. He presented with painless macroscopic hematuria for about three weeks, related to physical effort and sexual intercourse. No previous episodes or traumatic or inflammatory history was reported, and systemic examinations were normal.

**Figure 1:**
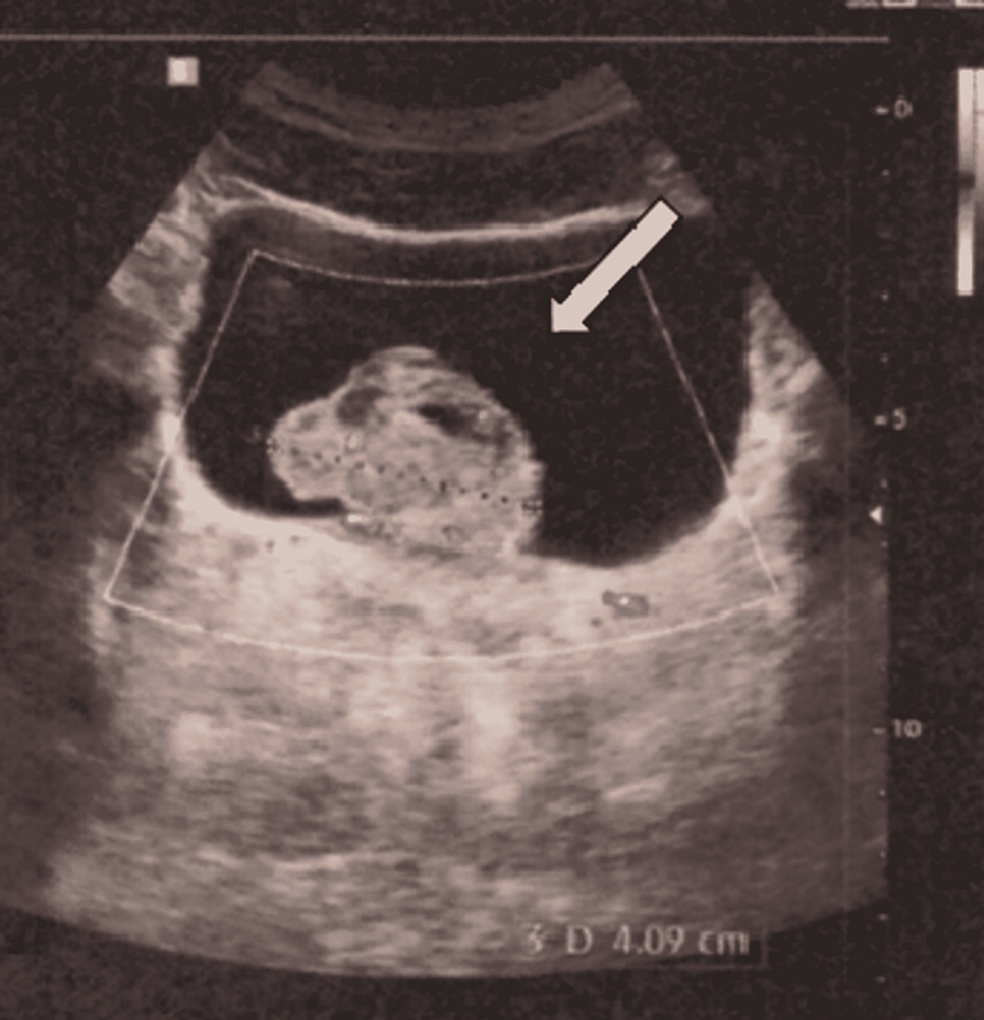
Ultrasonography showing a bladder lesion (white arrow) in the posterior wall with signs of neoplasia.

Ultrasound of the urinary tract was performed, showing bladder with a prominent vegetating lesion, with the presence of visible vascularity detected by color Doppler signal, measuring 3.3 x 3.4 x 4.1 cm, immobile to the change of position, in its posterior wall with signs of malignant neoplasm (Figure 1).

The patient underwent cystoscopy and transurethral resection of the bladder lesion, which had a sessile and friable aspect. The procedure was performed without complications and the patient was discharged on the first postoperative day.

**Figure 2:**
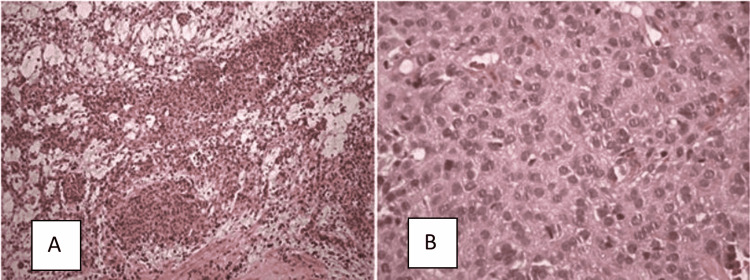
Histological section Hematoxylin-eosin staining showing fragments of the bladder affected by neoplasm forming solid groups and a rich vascular network in between (A – 100x), with round and ovoid cells, with slightly eosinophilic cytoplasm (B – 400x).

The individual returned to the office 15 days later, complaining of mild dysuria, which lasted for five more days. The hematoxylin-eosin staining of the biopsy showed round and ovoid cells, with slightly eosinophilic cytoplasm and monotonous nuclei forming solid spiral clusters with very rare mitosis figures (Figure 2). The material was sent for immunohistochemical analysis, which revealed negative S-100 protein, with epithelial membrane antigen (EMA) and claudin-1 positivity. In the morphological context, the positivity of these two antibodies corroborates the diagnosis of epithelioid extraneural perineurioma pattern (Figure 2 and Table 1).

**Table 1:** Immunohistochemical panel showing positivity for epithelial membrane antigen (EMA) and claudin-1

Antibody	Clone	Result
Cytokeratin 40, 48, 50 and 50.6 kDa	AE1/AE3	Negative
GATA-3, transcription fator in breast	C50-823	Negative
Chromogranin A	DAK-A3	Negative
Smooth muscle actin	1A4	Negative
Desmin (intermediate filament of muscle cell)	D33	Negative
Protein S-100	Polyclonal	Negative
Epithelial membrane Antigen (EMA)	E29	Positive
Claudin-1	Polyclonal	Positive
GLUT-1	SPM498	Negative
CD34	QBEnd 10	Negative

Six months after the procedure, the patient was asymptomatic, and a pelvic magnetic resonance imaging was requested for follow-up, which did not show any bladder or pelvic modifications.

## Discussion

First described in 1978 by Lazarus and Trombetta, perineuriomas are tumors derived from perineural cells and belong to the category of benign nerve sheath neoplasms along with neurofibromas and schwannomas [1,2]. The perineurium is part of the supportive structure of the nerve trunk; it is a lamellar structure formed by one to six rows of flattened cells and a variable amount of connective tissue that surrounds the nervous fascicles constituted by axons, Schwann cells, and endoneurium, functioning as mechanical protection and individual blood-brain barrier for each fascicle [1,3,4].

Perineuriomas are classified into two forms: intraneural and extraneural. Both share ultrastructural and immunohistochemistry profiles, being distinguishable in clinical and histopathological presentations [1,4]. Intraneural perineuriomas resemble schwannomas, like fusiform masses along the great nerves. The extraneural ones are subdivided into sclerosing and soft tissue perineurioma [4-5]. The former has outlines of a benign neoplasm, vertically oriented, relatively symmetrical, and well defined, but not encapsulated. The latter has a variable size from millimeters to centimeters, with prevalence in the subcutaneous tissues of the extremities, the scapular or pelvic girdle, and the trunk; however, 30% of the cases involve deeper soft tissues and rare cases are from dermal location [1].

As for the histological pattern, the soft tissue perineurioma (STP) is formed by elongated spindle cells with tapered nuclei, light eosinophilic cytoplasm, indistinct cell boundaries with elongated and extremely thin bipolar cytoplasmic processes found at least focally, presenting great variability in architectural patterns, among them, storiform, fascicular, reticular, plexiform, infiltrative and myxoid. The sclerosing perineurioma (SP) presents the sclerotic stroma with a large amount of collagen, it is composed of cells that are arranged between the collagen bundles in small nested rows, trabecular or spiral. These cells have two distinct shapes, a fusiform and wavy and another rounded and epithelioid with evident cytoplasm, both of which may be present in the same lesion. The nuclei are small and hyperchromatic and the nucleus is indistinct. Mitosis figures are rare or absent and there is no nuclear pleomorphism. Some lesions have thin-walled dilated blood vessels and nerve fascicles are present in the midst of the neoplasm [1,4].

In clinical practice, an accurate diagnosis will require an experienced pathologist with or without the aid of immunohistochemical profiling [4-5]. In intraneural perineuriomas, weakness, as well as sensory and mass loss, are the most common complaints. On the other hand, extraneural perineuriomas generally affect middle-aged adults, with a slight female preponderance and without neurological symptoms [1,5,6].

Using immunohistochemistry, perineuriomas are positive for epithelial membrane antigen (EMA) and negative for S100, a family of calcium-binding proteins and neurofilaments, which differentiates them from schwannomas. Other non-specific antigens frequently expressed by these cells are Glut-1, claudin-1, collagen IV, and laminin [5-6].

The treatment of perineuriomas consists of surgical excision and the recurrence is rare [5-6].

## Conclusions

This case describes an extraneural perineurioma occurring in the bladder, a location that to our knowledge has not yet been described in the literature. Therefore, it should be included in the differential diagnosis during the analysis of resections of bladder tumors, mainly in young adults, in whom the presentation of a bladder tumor is uncommon.
